# Pathophysiological hypotheses of the triad in abusive infant shaking: A systematic review and analysis of corroborated cases

**DOI:** 10.1016/j.fsisyn.2025.100618

**Published:** 2025-06-30

**Authors:** Ingemar Thiblin, Carl Johan Wingren, Jacob Andersson Emad, Fredrik Tamsen

**Affiliations:** a Department of Surgical Sciences, Forensic Science, Uppsala University, Uppsala, Sweden; b Department of Forensic Medicine, Section of Forensic Pathology, University of Copenhagen, Copenhagen, Denmark

**Keywords:** Abusive head trauma, Subdural hemorrhage, Retinal hemorrhage, Encephalopathy, Shaken baby syndrome, Pathophysiology, Chronic subdural hematoma

## Abstract

Subdural hemorrhage, retinal hemorrhages, and encephalopathy are associated with the medical diagnosis of abusive head trauma. These findings have also been observed in children exposed to admitted or witnessed shaking. There are various suggested mechanisms behind these findings. One mechanism is exclusive to intentional shaking, while the other suggested mechanisms are compatible with both intentional and accidental violence as well as an underlying illness.

We performed a systematic literature review of case reports on triad findings with subsequent analysis on the empirical consequences of three mechanistic hypotheses: (1) the outcome components arise independently following acceleration-deceleration forces during shaking; (2) the outcome components are partially dependent and caused by pathophysiological mediators following hypoxia caused by damage to the brainstem or cervical spinal cord by shaking; (3) the outcome factors are partially dependent and are caused by re-bleeding triggered by shaking in chronic subdural hematoma.

From a total of 9628 articles, we identified twelve publications including in total 100 cases that met the inclusion criteria. We identified no sufficiently detailed case report, but nine cases had information that allowed for tentative testing of the hypotheses. Three cases had findings consistent with that triad findings are partially dependent and related to chronic subdural re-bleeding (hypothesis 3), whereas no case provided support for the other hypotheses. Thus, published cases do not provide the information needed to understand the mechanism underlying triad findings in infants subjected to shaking.

## 1 Background

The concept of shaken baby syndrome (SBS), linking infant shaking with a constellation of findings: 1) subdural hemorrhage (SDH), 2) retinal hemorrhages (RH), and 3) encephalopathy constituted by either symptoms suggestive of traumatic brain injury (TBI) such as seizures or loss of consciousness or findings e.g., cerebral edema [1,2] was suggested in the 1970s. The three components, sometimes referred to as the triad, were assumed to be independent of each other with a common underlying causal mechanism in the form of acceleration and deceleration forces [2]. These forces were supposed to cause tear damage to bridging veins with accompanying subdural hemorrhage, traction forces between the vitreous and the retina with accompanying retinal hemorrhages, and tear damage to axons in the brain with accompanying neurological symptoms (encephalopathy). This mechanistic hypothesis was questioned for the first time in 1987 based on a biomechanical model that indicated that shaking does not generate sufficient forces to cause bridging vein damage or diffuse axonal injury [3]. Later biomechanical studies also supported that conclusion, i.e., have not been able to substantiate shaking as a cause of subdural hemorrhage [4,5]. However, one study indicates that end point impact, i.e., that the head hits the chest during shaking could generate forces that cause bridging vein damage [6].

In 2009, the American Academy of Pediatrics recommended that a mechanism-neutral term replace SBS [7]. The proposed term was abusive head trauma (AHT), which includes shaking as well as blunt force head trauma, or a combination thereof. It is well known that blunt force head trauma can cause subdural hemorrhage. In infants, this is supported by observed, in two cases filmed, household fall accidents with occipital impact [[8], [9], [10], [11]] and in one case face impact [12]. RH were present in 12 of the 13 cases presented in these case reports. Atkinson et al. hypothesize that this could be the result of a rotational component involved with occipital impacts [10], whereas Gardner suggest that the RH may be secondary to intracranial pathology [9].

In the early 2000s, a neuropathological study, described in two articles, demonstrated hypoxic brain injury but not TBI in children with triad findings and which were presumed to have been shaken with no evidence of blunt force head trauma, e.g., skull fracture [13,14]. Some children had axonal injuries in the brain stem, and it was proposed that shaking gives rise to damage to the brainstem/medulla, which causes respiratory deficiency and hypoxia with brain swelling and raised intracranial pressure [15]. This hypothesis is also compatible with the different components of the triad being independent of each other, but necessary assumptions are that shaking alone can cause subdural hemorrhage and that retinal hemorrhages are caused by traumatic acceleration/deceleration forces having effects directly on structures in the eye.

Based on these neuropathological observations and anatomical and physiological considerations, an alternative basic mechanism for the origin of the triad has been proposed. This hypothesis assumes that hypoxia underlies the triad in such a way that hypoxia leads to brain edema with venous congestion and raised intracranial pressure, and that the venous congestion in turn gives rise to intradural bleeding, that breaks through to the subdural space, and retinal hemorrhages. According to Geddes et al., the starting point could be axonal damage in the brainstem related to shaking [15]. In this model, one can hypothesize that the initial apnea is followed by other encephalopathy symptoms, e.g., seizures.

Another hypothesis assumes that retinal hemorrhages and encephalopathy are secondary to an acute subdural re-bleed as a complication of chronic subdural hemorrhage or healing subdural membranes which may be birth-related [16]. There is also evidence of subdural hematoma as a complication to benign external hydrocephalus (BEH) [[17], [18], [19], [20], [21], [22], [23], [24], [25], [26], [27], [28]]. BEH are often asymptomatic, but there are case reports indicating that BEH with subdural hematoma can be symptomatic presenting with encephalopathy and retinal hemorrhages [[29], [30], [31]].

The original hypothesis on SBS, acceleration-deceleration forces as the common underlying mechanism for triad findings, not only lack support from biomechanical studies, but the majority of the studies establish the exposure to shaking based on the presence of triad findings with or without other findings consistent with physical abuse [32]. By doing so, the acceleration-deceleration mechanism is taken for granted, which means that the association is supported by circular reasoning and not empirical observation [[32], [33], [34], [35]].

On the other hand, the evidence for the alternative hypotheses is also weak. Injury to the brainstem or cervical cord has primarily been described in deceased infants where shaking is assumed [13,36], and the mechanism is only exceptionally confirmed by confession [37]. Such injury in surviving infants with a concomitant subdural hemorrhage (assumed to be inflicted) has not been described apart from subdural blood surrounding the spinal cord [36,38,39].

When it comes to re-bleeding in chronic subdural hematomas, it is usually unclear what caused the older SDH, with the exception for BEH that can be identified by clinical and radiological findings, independent of the triad. There is evidence for SDH, with or without RH, as an asymptomatic or symptomatic complication to BEH [17,29,30,40,41]. However, spontaneous collapse in combination with RH is not scientifically established as this has, to our knowledge, only been described once as observed by an independent person [31].

In a forensic perspective, it is problematic that the mechanism behind triad findings is unclear. As the task of the forensic pathologist is to determine if a medical finding is an injury or not, and in the case of injury, assess whether the injury is compatible with the course presented.

Usually, assessing the mechanism of injury does not cause any concern, as injuries following e.g., gunshots, sharp objects or blunt force trauma have highly characteristic appearances. Next is to assess how the injury was inflicted, e.g., through an accident or abuse. In this respect, violent shaking of an infant is unique; as such an act can only be intentional. The fact that the child cannot explain what has happened, means that the assessment must rely entirely on the interpretation of the medical findings, where typical traumatic markers such as fractures or skin lesions often are absent. This further underlines the importance of understanding the mechanism behind the triad findings.

The purpose of this review is twofold; (1) to assess the quality of publications where exposure to shaking is established independently of findings, and (2) to test suggested mechanism behind triad findings based on assessment of cases selected for sufficient information on exposure and medical findings.

## 2 Methods

### 2.1 Overall strategy

The first step was to select articles that presented at least one case that met the criteria for inclusion.

The second step was to map the occurrence of a set of variables on exposure, medical findings, and previously suggested vulnerability factors at the individual level (Appendix 1). This step led to exclusion of cases that lacked key information on exposure or medical findings. The results before and after exclusion address the first research question of this study, which is to examine the extent to which information about exposure and outcomes is reported.

The third step was to rank the reporting of the remaining cases according to our quality criteria: sufficient data on exposure and outcome allowing for testing the compatibility of the cases with the theoretical empirical consequences of the three different mechanism hypotheses. The criteria for inclusion and exclusion are reported in section 2.6.

### 2.2 Study eligibility criteria

#### 2.2.1 Study design

We included observational studies, including case reports and case series, if the exposure shaking was defined independently of medical findings, i.e., cases of witnessed or admitted shaking. If the exposure was defined, fully or partly, by the medical findings the case was excluded (risk for circularity bias).

#### 2.2.2 Participants

The target population included infants up to one year of age at the point of medical work up.

### 2.3 Literature search

We searched PubMed, Cochrane library, and Ovid Embase in November 2022 using the same search terms applied by the Swedish Health Technology Assessment Authority (SBU) in a systematic review on the diagnostic accuracy of the triad for detecting infant abuse by isolated shaking (Appendix 2). The searches were restricted to publications in English. A research librarian at the Uppsala University Library carried out the search. The identified references were imported into Rayyan for further screening.

### 2.4 Study selection

Titles and abstracts were screened by two reviewers for relevance. Discrepancies were resolved through discussion or by inclusion of the discrepant record to full-text assessment. Following the title and abstract screening, each potentially relevant article was read in full text and independently assessed for inclusion by two reviewers, with any discrepancies resolved through consensus and, if necessary, by referral to a third reviewer.

### 2.5 Data extraction

A data extraction form was developed in a custom-built Microsoft Excel database. For each case, we extracted information on sex of the infant and the suspected perpetrator, age of the infant, country, exposure, outcomes, and vulnerability factors. Data extraction was carried out by two reviewers; discrepancies were resolved through discussion.

### 2.6 Inclusion and exclusion of individual cases

The basic inclusion criteria were age up to one year and an explicit statement of shaking either by a witness or the perpetrator, and some kind of intracranial lesion or retinal hemorrhage. There was no requirement that the statement had to be made spontaneously before medical findings were presented, and confessions during police interrogation were accepted even if the interrogation procedure was not described.

Criteria for exclusion were:

• A statement of blunt force head trauma or findings indicative of such trauma (head skin lesions or skull fracture).
• A statement of strangulation.
• Shaking after debut of symptoms or unclear when the shaking took place in relation to the debut of symptoms.

### 2.7 Quality assessment of individual cases

Since this review is based on case reports or case series, which do not provide any evidence of causality, we did not perform a risk of bias assessment. Instead, we assessed the quality of the case reports based on the level of details provided regarding the exposure and the outcome. The rational for this is that the aim was not to investigate the evidence for a generalizable causation between isolated shaking and triad findings, but to explore the possible mechanism in cases selected to allow for a reasonable assumption of isolated shaking as the cause of the findings.

There is no established tool for quality assessment of case reports on exposures of non-intervention type. For that reason, a model for quality assessment of the case reports was constructed.

Different criteria were used for witnessed and admitted abuse, respectively. If there was both a witness and a confession of the shaking episode, the case was treated as witnessed. If it appeared that the abusive act was either witnessed or confessed, but it was not possible to discern between these two alternatives, the case was treated as admitted abuse.

In the final analysis based on the cases reaching excellent, high or moderate quality, the witnessed and admitted cases were merged into a single database.

#### 2.7.1 Quality assessment of exposure

##### 2.7.1.1 Quality grading and exclusion criteria of witnessed exposure

A. Excellent quality

The highest degree of quality for a witnessed event is documentation by filming. The rational for this is that the event is proven and that it allows for a subjective assessment of the force employed.

B. High quality

In a previous publication on witnessed fall from a low height, it is stated that at least two persons must have witnessed the event [42]. In another publication the definition is that at least two persons, or a person who was not involved in the child's care must have witnessed the event [43]. Based on these definitions, in the present review, the second highest degree of quality is defined as the event being witnessed by at least two closely related persons (e.g., partner or relative) or one independent person (e.g., neighbor).

C. Moderate quality

The event was witnessed by a single close relative (e.g., partner or parent). The rational for this level is that a conflict between partners or relatives might affect the trustworthiness of such a witness.

D. Low quality

If it is unclear who witnessed the event, the degree of quality is set to “Low quality”, resulting in exclusion of the case from the quantitative analysis.

##### 2.7.1.2 Quality grading and exclusion criteria of admitted exposure

A scale for grading the quality of admissions of AHT was employed in 2022 in a review article [44]. The ranking is based on the level of details provided in the admission. We employed that scale with some modifications.

If the criterion regarding the temporal relationship between the exposure and the onset of symptoms is met, with the additional criterion that the admission was spontaneously given to the hospital staff before the demonstration of any findings, it is regarded as A. Excellent quality. For the other levels the ranking scale by Edwards et al. [44] was used without further modification, with B ranked as “High quality”, C as “Moderate quality”, and D as “Low quality” leading to exclusion. The rational for adding the criterion of spontaneously admitted event to get the highest degree of quality is that the mechanism of shaking cannot have been suggested to the perpetrator because of medical findings, and that the confession cannot be the result of coercion during the legal investigation. Thus, our grading scale is defined as:

A. The mechanism of injury (shaking, impact, shaking/impact, other) is reported, and all details of the event are provided (antecedent circumstances, aftermath of the event, and the motivation of the confessor). The admission was spontaneously given before the demonstration of any findings.
B. The mechanism of injury is reported and two details among antecedent circumstances, aftermath of the event (here we include effect, e.g., seizures), and the motivation of the confessor are provided.
C. The mechanism of injury is reported and only one detail is provided.
D. The mechanism of injury is reported, but no detail is provided.

If there was no information concerning the time between the exposure and the debut of symptoms, the case was graded as D irrespective of the extent of occurrence of the other parameters. The reason for this is that the mechanistic hypotheses cannot be tested without knowing the exposure-symptom interval.

#### 2.7.2 Quality grading and exclusion criteria of reported intracranial pathology

A. Excellent quality

For the highest degree of quality (Excellent quality), there should be a detailed description of the neuroradiological and/or neuropathological findings including:

• a dating parameter, e.g., the density of an SDH on a CT scan or the presence of siderophages in histology
• the exact location and size (including thickness of a subdural hematoma) of the lesion
• explicit interpretation of brain edema as related to hypoxic brain injury or TBI
• verbal statement on expansive effect in the case of subdural hematoma
• at least one picture from neuroimaging/gross pathology.
B. High quality

For the second highest degree of quality (High quality), the same criteria are valid as in A, except that a picture from neuroimaging is not included. If the verbal description is incomplete and there is a neuroimaging image that fully compensates for the missing information, the criteria for “High quality” are met.

C. Moderate quality

The verbal description is incomplete but includes the type of lesion, e.g., SDH, subarachnoid hemorrhage, brain edema and, when it comes to SDH, a dating parameter.

No picture from neuroimaging is included in the criteria.

D. Low quality

The type of lesion is stated but no further information is provided.

#### 2.7.3 Final assessment of quality

For each case the two dimensions of quality; ascertainment of exposure and description of findings were combined, and the lowest score defined the quality. Subsequently, the combined assessment was transferred to final assessment of quality in the following manner:

AA: Excellent quality.

AB, BB: High quality.

AC, BC, CC: Moderate quality.

AD, BD, CD, DD: Low quality.

#### 2.7.4 Ad hoc assumptions

Because several variables were reported in different manners, we made several assumptions.

• If bruises were reported without anatomical specification, we assumed that they were on another part of the body than the head region.
• If there were bruises or “marks” in the head region, these were assumed to be the result of pinching or scratching if it was made clear in the methodology section that cases with signs of blunt force head trauma had been excluded.
• If a CT or MR of the head had been performed and there was no explicit comment on intracranial findings, we assumed that no intracranial pathology was present. Similarly, if fundoscopi had been performed and there was no explicit comment on pathological findings in the eye, we assumed that no eye pathology was present.
• If it was clear that the case had been subjected to a clinical examination without mentioning of bruises or skin lesions on the head, we assumed that such findings had been ruled out, even if not explicitly declared.
• If there was no explicit statement of the shaking preceding symptoms, we regarded a statement of shaking out of frustration as shaking preceding symptoms.
• Crying was not considered as a symptom of encephalopathy.
• If brain contusions were reported without mentioning of a skin lesion or a skull fracture, the contusions were regarded as possibly related to the proposed acceleration-deceleration mechanism caused by shaking, thus it was not registered as a sign of blunt force trauma.
• If the infant had been the subject of an autopsy and there was no explicit comment regarding sinus thrombosis and it was not reported that the sinuses had been examined, “Presence of sinus thrombosis” was registered as “Unclear”.

### 2.8 Analysis of provided information

#### 2.8.1 Analysis of variable reporting

The first study question (Does the study include sufficient data to allow for analysis with respect to shaking as the mechanism behind the triad findings in witnessed or admitted cases of isolated shaking?) included all cases in the twelve identified articles with a maximum age of one year in the first selection irrespective of the quality grading. In this analysis, the frequency of reporting of variables is regarded as outcome.

#### 2.8.2 Analysis of mechanism of findings

For the second study question regarding the different mechanistic hypothesis, only cases reaching excellent, high or moderate quality were included.

##### 2.8.2.1 Predicted empirical consequences

Based on the theoretical considerations of the three main mechanism hypotheses, expected empirical consequences were defined.

**Fig. 1:**
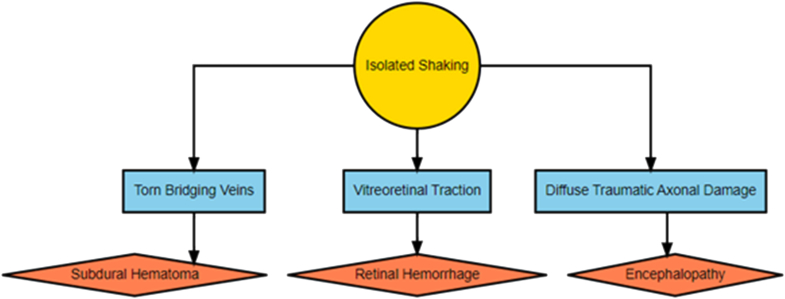
The acceleration-deceleration hypothesis. Yellow signifies underlying cause, blue signifies mediators, and red signifies diagnostic markers.

The initial shaking hypothesis assumes direct mechanical effects; that acceleration - deceleration forces give rise to SDH through damage to bridging veins, retinal hemorrhage through traction forces between the vitreous and the retina, and neurological symptoms such as vomiting, seizures, loss of consciousness and apnea related to TBI (Fig. 1). From this the following empirical consequences are expected:

• The SDH is acute and anatomically related to bridging veins
• The RHs are acute
• There is no evidence of blunt force head trauma other than possible intracranial injuries that can be inflicted by shaking as well
• The neurological symptoms appear in immediate connection with the exposure
• If apnea/respiratory compromise has taken place there may be secondary hypoxic brain injury
• There may or may not be injuries on other regions than the head.

**Fig. 2:**
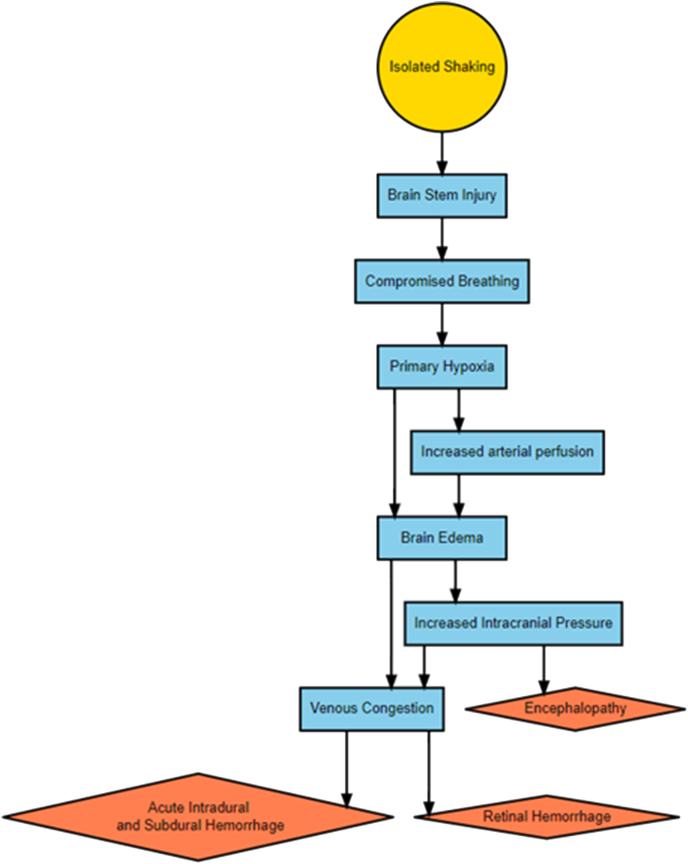
The brainstem or cervical spine injury-hypoxia hypothesis. Yellow signifies underlying cause, blue signifies mediators, and red signifies diagnostic markers.

The hypoxia hypothesis assumes that injury to the lower brainstem or the cervical spine is the first step and that that hypoxia is the second step in a pathophysiologic chain that causes all components of the triad (Fig. 2). A central aspect of the hypoxia hypothesis is that the hypoxia leads to congestion in an intradural vein plexus with leakage of blood causing a subdural blood collection [45]. This plexus is thought to disappear at approximately six months of age [16]. From the hypoxia hypothesis the following empirical consequences are expected:

Hypoxia caused by brainstem or cervical spine injury related to shaking.

• The subdural hemorrhage is acute, thin and widespread
• The retinal hemorrhages are acute
• Hypoxic brain injury is demonstrated on neuroimaging or neuropathology
• There are signs of raised intracranial pressure
• There is no evidence of blunt force head trauma other than possible intracranial injuries that can be inflicted by shaking as well
• The infant is six months old or younger
• There is a traumatic brain stem or cervical spine injury
• There may or may not be injuries on other regions than the head

**Fig. 3:**
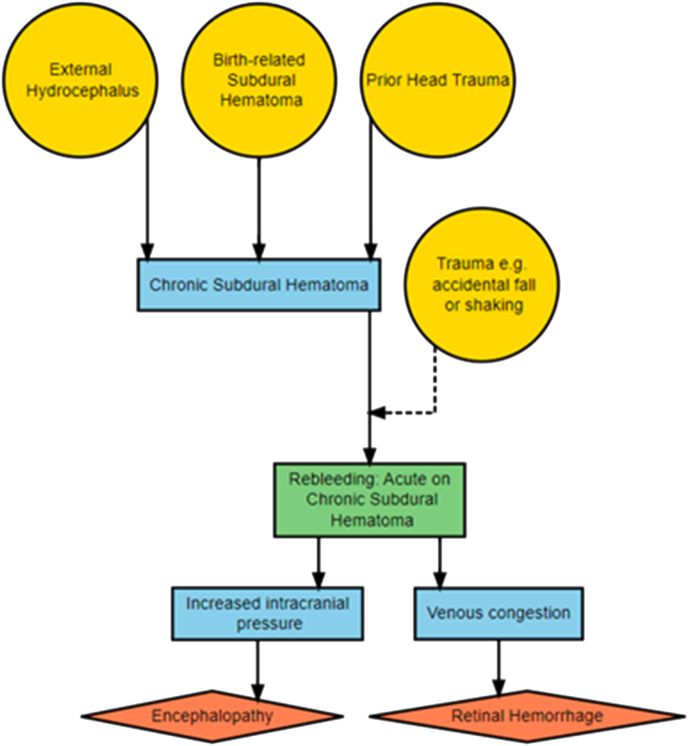
The re-bleeding hypothesis. Yellow signifies underlying cause, blue signifies mediators, red signifies diagnostic markers, and green signifies both mediator and diagnostic marker.

The re-bleeding hypotheses is that an acute bleeding in a chronic subdural hematoma triggers a pathophysiological cascade that causes encephalopathy and retinal hemorrhage (Fig. 3). The cause of the re-bleeding may be isolated shaking. From the re-bleeding hypothesis the following empirical consequences are expected:

##### 2.8.2.2 Re-bleeding related to isolated shaking

• The subdural hemorrhage is both chronic and acute (mixed density)
• The retinal hemorrhages are acute
• If apnea/respiratory compromise has taken place there may be secondary hypoxic brain injury
• The symptoms might be both acute and delayed
• There are signs of raised intracranial pressure
• There is no evidence of blunt force head trauma other than possible intracranial injuries that can be inflicted by shaking as well
• There may or may not be injuries on other regions than the head

## 3 Results

### 3.1 Literature screening

**Fig. 4:**
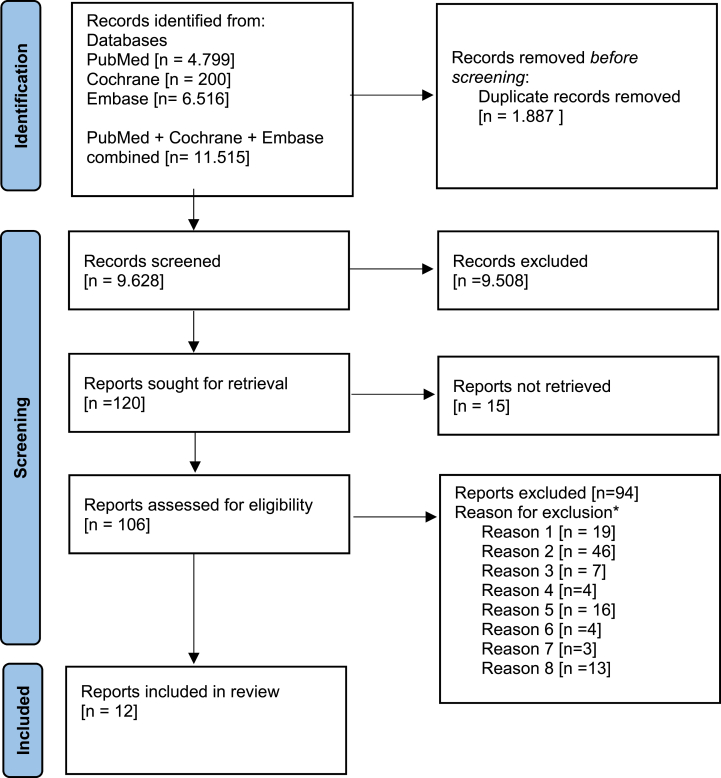
Flow chart of literature screening by PRISMA guidelines ∗Thirteen articles had more than one reason. Reason 1: Insufficient data, Reason 2: Not witnessed or admitted, Reason 3: Not shaking, Reason 4: Shaken after debut of symptoms, Reason 5: Unclear when shaking occurred, Reason 6: Review or Guidelines, Reason 7: No intracranial findings, Reason 8: Blunt head trauma.

After removing duplicates our searches identified 9628 articles. Title and abstract screening resulted in exclusion of 9.508 irrelevant records, leaving 120 records requiring full text assessment. Of these, 15 publications could not be obtained and 94 were excluded after full text assessment (Fig. 4, Appendix 3). We included 12 publications for data extraction.

### 3.2 Quality assessment of description of exposure and medical findings

The assessment for the quality in the description of the exposure and in description of the intracranial findings is presented in Table 1. In summary, no case reached excellent or high quality, 9 cases reached moderate quality, and 31 cases were rated as low quality. The reason for getting low quality was insufficient information on the neuroimaging findings in 9 cases, insufficient information on the exposure in 19 cases, and insufficient information on both neuroimaging findings and exposure in three cases.

**Table 1:** Quality assessment of exposure and intracranial findings.

Reference/Name of case	Quality Exposure	Quality Findings	Combined assessment	Reasons for concerns
*Witnessed shaking*				
[46]/Case 1	D	C	Low	Time to symptoms unclear
[46]/Case 2	D	D	Low	No information on symptoms
				No dating of SDH
[46]/Case 3	C	D	Low	No dating of SDH
[46]/Case 4	C	D	Low	No dating of SDH
[46]/Case 5	D	D	Low	No information on symptoms
				No dating of SDH
*Admitted shaking*				
[47]/Patient 2	D	C	Low	Time to symptoms unclear
[47]/Patient 3	D	A	Low	Time to symptoms unclear
[47]/Patient 6	D	C	Low	Time to symptoms unclear
[47]/Patient 7	D	C	Low	Time to symptoms unclear
[47]/Patient 8	D	C	Low	Time to symptoms unclear
[47]/Patient 9	D	C	Low	Time to symptoms unclear
[47]/Patient 10	B	C	Moderate	
[47]/Patient 11	C	C	Moderate	
[47]/Patient 13	D	C	Low	Time to symptoms unclear
[47]/Patient 15	D	A	Low	Time to symptoms unclear
[47]/Patient 16	C	C	Moderate	
[47]/Patient 18	D	C	Low	Time to symptoms unclear
[47]/Patient 20	D	C	Low	Time to symptoms unclear
[47]/Patient 21	C	C	Moderate	
[47]/Patient 22	D	C	Low	Time to symptoms unclear
[47]/Patient 23	B	C	Moderate	
[47]/Patient 24	D	C	Low	Time to symptoms unclear
[47]/Patient 25	B	C	Moderate	
[47]/Patient 27	B	C	Moderate	
[47]/Patient 28	D	C	Low	Time to symptoms unclear
[47]/patient 29	D	C	Low	Time to symptoms unclear
[48]/Case report	D	A	Low	Time to symptoms unclear
[49]/Case report	D	A	Low	Time to symptoms unclear
[50]/Case 1	D	B	Low	Time to symptoms unclear
[51]/Case 1	A	D	Low	No dating SDH
[51]/Case 2	A	C	Moderate	
[51]/Case 3	A	D	Low	No dating SDH
[51]/Case 4	C	C	Moderate	
[52]/Case 1	D	C	Low	Time to symptoms unclear
[53]/Case 2	C	D	Low	Vague description of SDH
[54]/Case 2	C	D	Low	No dating SDH
[55]/Case 2	D	D	Low	Time to symptoms unclear No dating SDH
[55]/Case 3	C	D	Low	No dating SDH
[56]/Case 4	C	D	Low	No dating SDH
[57]/Case 1	A	D	Low	No dating SDH

### 3.3 Overall reporting of study variables

#### 3.3.1 Witnessed cases

The five witnessed cases that fulfilled the inclusion criteria all presented with information on intracranial findings, age, sex, trauma mechanism, signs of blunt head trauma, retinal hemorrhage, skin lesions, and fractures [46]. Dating of the subdural hematoma was provided in one case. Encephalopathy was considered in all five cases and time to symptoms was described in three cases.

Information concerning gestational age was provided in one case. Data on delivery, multipara, and neonatal conditions were not provided in any case.

#### 3.3.2 Admitted cases

In the initial 95 cases that were evaluated by systematic registration of the 63 study variables (Appendix 1), age was by definition (inclusion criterion) reported in all cases, and most cases presented sex. Information on exposure was missing in 12 % of the cases and a minority of the cases had information on when in the process an abusive act was admitted. The most central medical findings (see medical findings in Table 2) were reported in more than 80 % of the cases with the exception for dating of subdural hematoma, which was only stated in 60 % of the 75 cases having such a lesion. Potential vulnerability factors such as prematurity and small for gestational age were reported in a minority of the cases. Detailed information is provided in Table 2.

**Table 2:** Frequency of basic variable reporting (explicitly commented if not stated otherwise) in cases selected for systematic evaluation before applying exclusion criteria (n = 100) and after applying exclusion criteria (n = 42).

Variable	Number of cases with information on the variable (positive or negative) before exclusion (n = 100)	Number of cases with information on the variable (positive or negative) after exclusion (n = 42)
General descriptive data		
Age	100 (by inclusion criterion)	42(by inclusion criterion)
Sex	90 (90 %)	39 (93 %)
Exposure		
Trauma (any kind)	89 (89 %)	42 (by criteria)
Signs of blunt force trauma (skin injury/fracture)	98 (98 %)	42
When abusive trauma was admitted (only admitted cases)	81 (85 %)	37
Time between exposure and symptoms	24 (39 %)	19 (45 %)
Only cases with exposure preceding symptoms considered (n = 62, n = 42)		
Sex of perpetrator	61 (61 %)	33 (79 %)
Medical findings		
Presence of intracranial lesion	100	42
Dating of subdural hematoma	46 (58 %)	31 (76 %)
Only cases having SDH considered (n = 80, n = 41)		
Encephalopathy	95 (95 %)	42
Retinal hemorrhage	89 (89 %)	41(98 %)
Fracture	97 (97 %)	42
If x-ray performed (n = 97), no mentioning of a fracture regarded as indirect information of negative.		
Bruises on other body regions than the head	95 (95 %)	42
Vulnerability factors		
Gestational age	42 (42 %)	25 (60 %)
Multipara	9 (9 %)	1 (2 %)
Type of delivery	11 (11 %)	2 (5 %)
Birth weight or SGA/LGA	8 (8 %)	1 (3 %)
Perinatal disease	7 (7 %)	1 (3 %)

After applying the exclusion criteria on the reporting of the individual cases in the selected articles, there was a clear increase in the proportion of cases having information on when the abusive act had been admitted, sex of the perpetrator, dating of the subdural hematoma, and on gestational age (Table 2).

More detailed information on the medical findings, e.g., presence of an expansive effect, signs of raised intracranial pressure, laterality of subdural hematoma, thickness of subdural hematoma and the extent of retinal hemorrhages was rare, and are reported in section 3.6. where the mechanistic hypotheses are tested.

### 3.4 Exposure and outcome

#### 3.4.1 Exposure

In one of the five witnessed cases, the observer was an independent person (a neighbor), while in the remaining four, the observer was the perpetrator's partner. Among the 95 admitted cases included for individual case assessment (before applying the exclusion criteria), four belonged to the category of spontaneous confession before the detection of any findings. In the remaining admitted cases, the confession of shaking was given after the detection of findings: during the legal investigation (n = 33), at the hospital (n = 1) or under unclear circumstances (n = 42). In 11 cases, there was no statement of abuse of any kind, and in three cases only blunt force without shaking was admitted. After applying the exclusion criteria, 37 admitted shaking cases remained. In 23 cases the confession was given during the legal investigation, and in the remaining 14 cases the circumstances of the confession were unclear.

#### 3.4.2 Medical findings

##### 3.4.2.1 Intracranial findings and retinal hemorrhage

There were intracranial findings (by definition) and extensive RH in all five witnessed cases of shaking [46].

In the final set of 37 admitted cases, all had intracranial findings (by definition) and 30 also hade retinal hemorrhage. In five cases retinal hemorrhage was not present and in two cases the eyes had not been examined. Detailed information on the retinal hemorrhages was provided in five of the 30 cases having retinal hemorrhage. In two of those, the hemorrhages were described as extensive.

##### 3.4.2.2 Encephalopathy symptoms

Symptoms indicative of encephalopathy were reported in three of the five witnessed cases, and in 30 of the 37 admitted cases. In 27 cases, only one type of symptom was reported, in four cases two symptoms and in two cases three symptoms. A delay in symptom presentation that ranged from >1 h to >3 h was reported in five cases in one article [47].

Details concerning the symptoms in the witnessed and admitted cases are provided in Table 3.

**Table 3:** Symptoms reported in 33 cases having information on symptoms. A = symptoms in direct connection with shaking, B = symptoms at admission to hospital, C = Unclear when symptoms appeared (but clear it was after exposure).

Reference/Case	Seizures	Vomiting	Affected breathing	Affected level of consciousness
Witnessed shaking				
[46]/Case 1	No	Yes (C)	Yes (C)	Yes (C)
[46]/Case 3	No	No	No	Yes (Limp) (A)
[46]/Case 4	No	Yes (A)	No	Yes (A)
Admitted shaking				
[47]/Case 1	No	Yes (B)	No	No
[47]/Case 3	Yes (B)	No	No	No
[46]/Case 6	Yes (A)	No	No	No
[47]/Case 7	Yes (B)	No	No	No
[47]/Case 10	Yes (B)	No	No	No
[47]/Case 11	Yes (A)	No	No	No
[47]/Case 13	Yes (B)	No	No	No
[47]/Case 15	Yes (B)	No	No	No
[47]/Case 18	Yes (B)	No	No	No
[47]/Case 19	No	No	No	Yes (B)
[47]/Case 20	Yes (B)	No	No	No
[47]/Case 21	Yes (B)	No	No	No
[47]/Case 23	Yes (B)	No	No	No
[47]/Case 24	Yes (B)	No	No	No
[47]/Case 25	Yes (B)	No	No	No
[47]/Case 27	Yes (B)	No	No	No
[47]/Case 28	Yes (B)	No	No	No
[49]/Case report	Yes (A,B)	No	No	No
[50]/Case 1	No	No	Yes (A)	Yes (A)
[51]/Case 1	No	No	No	Yes (A)
[51]/Case 2	No	No	Yes (A)	Yes (A)
[51]/Case 3	No	No	No	Yes (A,B)
[51]/Case 4	No	No	Yes (A,B)	Yes (A,B)
[52]/Case 1	Yes (B)	No	Yes (B)	Yes (A,B)
[53]/Case 2	Yes (A)	No	No	Yes (A,B)
[54]/Case 2	No	No	Yes (A)	No
[55]/Case 3	No	No	Yes (A)	Yes (A,B)
[56]/Case 4	No	No	No	Yes (A,B)
[57]/Case 1	No	No	Yes (A)	Yes (A)
Total number	19	3	8	14

##### 3.4.2.3 Subdural hemorrhage

The characteristics of subdural hemorrhage in the merged final dataset are reported in Table 4.

**Table 4:** Characteristics of subdural hemorrhage in five witnessed and 37 admitted cases irrespective of quality and in 9 cases reaching moderate quality.

Type of subdural hemorrhage	Acute	Chronic	Mixed density	Unilateral	Bilateral	Thickness >5 mm
Final dataset (n = 42)	Yes: 11	Yes: 2	Yes: 17	Yes: 5	Yes: 6	Yes: 0
	No: 20	No: 29	No: 14	No: 7	No: 6	No: 9
	Unclear: 11	Unclear: 11	Unclear: 11	Unclear: 30	Unclear: 30	Unclear: 33
Cases with moderate quality (n = 9)	Yes: 4	Yes: 0	Yes: 5	Yes: 1	Yes: 1	Unclear: 9
	No: 5	No: 9	No: 4	No:1	No:1	
	Unclear: 0	Unclear: 0	Unclear: 0	Unclear: 7	Unclear: 7	

##### 3.4.2.4 Intracranial findings other than subdural hemorrhage

The results regarding intracranial findings, besides subdural hemorrhage, in witnessed and admitted shaking (all levels of quality, n = 42 and moderate quality, n = 9) are presented in Table 5.

**Table 5:** Intracranial findings other than subdural hemorrhage in five witnessed and 37 admitted cases all levels of quality, and in nine cases of moderate quality. ∗If not commented regarded as negative.

Finding	Subarchnoid hemorrhage	Hypoxic brain injury	Brain edema	Traumatic brain injury (DAI, laceration, contusion)	Sinus thrombosis or cortical thrombosis	Signs of BEH	Raised Intracranial pressure
Final dataset (n = 42)	Yes: 4	Yes: 20	Yes: 6	Yes: 3	Yes: 1	Yes: 0	Yes: 1
	No: 38	No:15	No: 36	No:39	No: 0	No: 4	No: 0
	Unclear: 0	Unclear: 7		Unclear: 0	Unclear: 41	Unclear: 38	Unclear. 41
Cases with moderate quality (n = 9)	Yes: 0	Yes: 7	Yes: 1	Yes: 0	Yes: 0	Yes: 0	Yes: 0
	No: 9	No: 2	No: 8	No: 9	No: 0	No:	No: 0
	Unclear: 0	Unclear: 0	Unclear: 0	Unclear: 0	Unclear: 9	Unclear: 9	Unclear: 9

##### 3.4.2.5 Fractures and skin lesions

Two of the five witnessed cases had bruises on other regions than the head, one of those had bruises also at the forehead and left cheek. The same two infants also had rib fractures and CMLs. There were no cases with long bone fractures. One of the other infants had a red mark in the face and the remaining two cases had no findings besides intracranial lesion or retinal hemorrhage.

In the group of 37 admitted cases, rib fractures were described in two cases, CML was described in two cases, and long bone shaft fracture was described in one case. In one case there was both CML and a long bone fracture. Thus, 31 infants had no fracture. Skin wounds (lacerations, bruises, skin swelling) were reported in 6 cases. One of those also had rib fractures.

When merging the witnessed and admitted cases 27 out of 42 (64 %) had no findings besides intracranial lesions and retinal hemorrhages.

##### 3.4.2.6 Cervical findings

Cervical skeletal or ligament injury was not described in any case. In one case (deceased) there was a spinal cord hematoma not further described [47]. This infant had a mixed density subdural hemorrhage, hypoxic brain injury, bilateral retinal hemorrhage, and ecchymoses not further described. Another infant (deceased) had ischemic changes in the spinal cord (unclear at what level) [57]. This infant had subarachnoid hemorrhage over both cerebral hemispheres, organizing subdural hemorrhage over left hemisphere and left frontal area; loosely adherent to the falx, hypoxic brain injury and sinus thrombosis.

### 3.5 Other descriptive data

#### 3.5.1 Sex of infant and perpetrator

In total (both witnessed and admitted cases) 28 infants were male and 13 female. In one case the sex was not reported.

The perpetrator was male in 26 cases, female in seven cases and not reported in nine cases.

#### 3.5.2 Country

The five witnessed cases were from the USA. The majority of the admitted cases were described in two French studies (n = 23). Other countries were Australia (n = 4), Canada (n = 2), Germany (n = 1), Japan (n = 1), Spain (n = 1) and the USA (n = 5).

### 3.6 Analysis of hypothetical mechanisms

No case had sufficient information enabling analysis of all predicted consequences. However, nine cases provided information to allow for tentative testing of some of the predicted empirical consequences. In two cases there was a description of uni/bilaterallity of the SDH in addition to dating. The thickness or volume of the SDH was not reported in any case, nor the presence of an expansive effect. Pictures from the neuroimaging was not provided in any of the nine cases.

The findings and symptoms in these nine cases are summarized in Table 6.

Hypothesis of triad spectrum findings being independent of each other and having a common cause in acceleration-deceleration forces

**Table 6:** Cluster of symptoms and findings in 9 cases of admitted shaking having moderate quality of the reported data.

Reference/Case	Acute SDH	Mixed density SDH	HBI/Brain edema	TBI Observed	Immediate symptoms	RH any kind	Other findings
47/Patient 10	Yes	No	Yes (HBI)	No	No	Yes	No
47/Patient 11	Yes	No	Yes (HBI)	No	Yes	Yes	No
47/Patient 16	No	Yes	Yes (HBI)	No	Yes	Yes	No
47/Patient 21	No	Yes	Yes (HBI)	No	No	Yes	No
47/Patient 23	No	Yes	Yes (HBI)	No	Yes	Not investigated	No
47/Patient 25	No	Yes	Yes (HBI)	No	No	Yes	No
47/Patient 27	Yes	No	Yes (HBI)	No	No	Yes	No
51/Case 2	No	Yes	Unclear	No	Yes	Yes	Femur fracture
51/Case 4	Yes	No	Yes (BE)	No	Yes	Yes	No

No case met all the morphological criteria (acute SDH + TBI + retinal hemorrhage) for the hypothesis that SDH, TBI and RH are independent of each other and have the same underlying traumatic cause in the form of acceleration-deceleration forces during shaking. Four cases had an acute SDH, but there was not any information on bridging vein damage or the exact location of SDH in any case. One of the cases with acute SDH was reported to have immediate symptoms in connection to shaking. This case also had a hypoxic brain injury (Case 11 in Ref. [47]).

Hypothesis that hypoxia related to cervical cord injury causes all triad spectrum findings/symptoms

Cervical cord injury was not reported in any case. Acute SDH in combination with hypoxic brain injury and retinal hemorrhage was present in four cases.

Hypothesis that re-bleeding in chronic subdural hematoma possibly caused by shaking causes the other components of the triad

Four cases had a mixed density SDH, and hypoxic brain injury was reported in three of those. Retinal haemorrhages were present in three cases. In one case there was no eye examination. Signs of raised intracranial pressure was not commented upon in any case.

## 4 Discussion

### 4.1 Quality of case reports

The quality of exposure descriptions was low in most reports, entailing exclusion of most cases. Only exceptionally was there a more detailed description of the exposure, such as duration of the exposure or the immediate symptoms of the child. In a large percentage of the cases, information was missing on whether the shaking occurred before or after the onset of symptoms, and there were also cases where it was stated that the onset of symptoms occurred before the shaking, which is surprising, since a causal relationship obviously requires that the exposure precedes the effect. Perhaps a statement that is not compatible with the shaking-acceleration-deceleration hypothesis is generally regarded as the informant is not being trustworthy, and that the stated reversed temporal relationship of shaking and symptoms in these cases therefore is considered to be false.

The medical findings were also insufficiently reported in a majority of the cases. A consequence of this is that the attempt to analyse hypotheses for causation, based on theoretical mechanistic assumptions failed, mainly due to the absence of data on subdural hemorrhage spread and thickness, signs of increased intracranial pressure and expansive effect. Hence, a tentative analysis was performed based on the nine cases that had fairly detailed data, and where it was possible, but not clear, that these factors could correspond to the theoretical assumptions on causative mechanisms.

The reason for why the description of the exposure and the medical findings has flaws may be that the article authors do not consider that a more detailed description is needed or that the information in the underlying medical records is sparse. It should also be considered that case reports usually have the purpose of flagging a possible causal link and that a problematizing approach with attempts to falsify the put forward hypothesis is therefore usually lacking.

Commenting on possible predisposing factors for infant subdural hemorrhage such as prematurity, multipara and small for gestational age [58] was rare and it is not clear to what extent this is explained by non-presence of such factors or negligence of such factors among the article authors. From the perspective of forensic medicine such factors may be of importance in both witnessed/admitted abuse cases and cases with a history of no trauma or accidental trauma.

Regardless of why the reports are insufficient in several aspects, future studies and case reports should strive to describe the exposure, medical findings, medical work-up for predisposing conditions and potential vulnerability factors as accurately and extensive as possible. This is a prerequisite for being able to approach the key question - if triad findings are independent of each other with a common mechanical cause or if encephalopathy symptoms and retinal hemorrhages are secondary to the intracranial process.

### 4.2 Tentative analysis of proposed mechanistic hypotheses

#### 4.2.1 Acceleration-deceleration force hypothesis

Although no case with moderate quality of reported data had all predicted empirical consequences of the shaking-acceleration-deceleration hypothesis, one case had immediate symptoms, retinal haemorrhages and a not further described acute subdural hemorrhage. Since it was not further described, it is possible, but cannot be claimed, that it was the result of bridging vein damage. However, instead of the expected TBI, there was a hypoxic brain injury. The reason for not identifying any case fulfilling the predicted empirical consequences could be that there are missed cases, due to diagnostic limitations such as low sensitivity for diffuse axonal brain injury in neuroimaging [59]. Another possible explanation is that the hypothesis is wrong.

#### 4.2.2 Hypoxia via compromised breathing hypothesis

The hypothesis that hypoxia is the results of compromised breathing caused by cervical spine injury did not gain support from the data of the included cases. Only one case with documented cervical spine injury was identified. The injury was described as spinal cord hemorrhage without further specification. It is therefore unclear whether it was a parenchymal-, a subarachnoid-, subdural-, or an epidural hemorrhage. The fact that cervical injuries were missing in most cases may be due to that they were not present or that they were missed. In only one of the reports, on a case without cervical injury, the finding was explicitly negated, indicating that the neuroimaging does not always include these structures in the assessment. Furthermore, neuroimaging has a limited sensitivity for detecting traumatic axonal injury in the brain [59], so it seems reasonable to assume that this is also the case when it comes to injuries in the cervical cord.

Considering that at least 7 of the 11 infants with an acute SDH (from the final dataset including all levels of quality) had a hypoxic brain injury and only one of those had a concomitant TBI (brain contusions), it could be that hypoxia rather than direct mechanical forces mediates the outcome. This possibility could not be further evaluated, due to the lack of details in the description of the subdural effusions.

In the article by Adamsbaum et al. [47], hypoxia was systematically reported and it was noted in 23 of the described 29 cases (including children older than one year and cases with indication of head impact). In a cluster analysis based on children selected for acute head injury in publications of diagnosed AHT, two clusters appeared: one driven by brain hypoxia/ischemia and the other by skull fracture and epidural hematoma. Hypoxia/ischemia was associated with respiratory compromise, encephalopathy, subdural hemorrhage or fluid collection, retinoschisis, and physician-diagnosed abuse [60]. Although hypoxia seems to be strongly associated with triad findings, it is not possible to determine if hypoxia is the underlying cause of the other findings or if hypoxia is the result of a preceding traumatic intracranial process. Thus, even if triad findings and symptoms are associated with hypoxic brain injury and not TBI, it could be that the hypoxic changes are related to a traumatic meningeal hemorrhage resulting in seizures and/or respiratory compromise. If so, triad findings would be non-specific with regard to type of trauma, as indicated in a recent study on a prospectively gathered material [61].

An explanation for the nearly total absence of neck injuries among the case reports in this review may be that shaking with grips around the shoulders or chest does not generate sufficient force to cause cervical injuries. This interpretation may seem to go against the results of a biomechanical study indicating that greater forces are required to cause subdural hematoma than is needed for cervical spinal cord injury in infants [62]. However, that study has been criticized for incorrect calculations [63]. At the same time, there is no study showing that greater forces are required to cause damage to the cervical spine/spinal cord than traumatic subdural hemorrhage. As this seems to be a key question, the need for further studies on this issue is urgent.

#### 4.2.3 Re-bleeding in chronic subdural hematoma hypothesis

The hypothesis that shaking triggers a re-bleeding in a chronic/subacute subdural hematoma requires that the subdural hematoma have both an old and a recent component. This criterion was met in four of the nine cases ranked as having moderate quality. The three who had been examined with fundoscopy had retinal hemorrhage, and in three cases a hypoxic brain injury had been diagnosed.

Subdural effusion with components of different age was also common in the final dataset of admitted shaking including all levels quality [n = 37] where at least 43 % had an effusion with components of different attenuation.

Re-bleeding in a chronic subdural hematoma may appear spontaneous or following minor trauma. The source of the bleeding is rupture of thin vessels in neomembranes or rupture of stretched cortical veins [64]. Thus, it seems reasonable to assume that shaking may trigger a re-bleed even if the force is not sufficient to cause bridging vein damage or neck injury in a healthy infant. In such a scenario the encephalopathy and retinal bleeds could be secondary to a suddenly raised intracranial pressure with venous congestion. Due to the sparse reporting of the presence, or non-presence, of raised intracranial pressure in the study material, this possibility could not be further evaluated.

#### 4.2.4 Concluding remarks on the mechanism hypotheses

Taken together, the retrieved data on the association between admitted or witnessed shaking and triad findings, indicate that shaking may cause the triad in the form of an acute on chronic/subacute SDH, where the symptoms and retinal hemorrhages may be secondary to the SDH. Furthermore, the data indicate that some kind of acute SDH related to hypoxia may also be the result of shaking. However, if so, the pathophysiological mechanism of how shaking could cause hypoxia is unclear. For instance, in the cases of non-lethal acute SDH and immediate symptoms, it cannot be ruled out that the hypoxic injury and SDH are secondary to a missed traumatic diffuse axonal injury.

### 4.3 Implications for forensic medicine

A key question in forensic interpretation is the specificity of the medical findings for abuse. The issue of specificity is brought to a head in the case of infants, as it is not unusual for the interpretation of medical findings to constitute the entire evidence in court. In general, abuse-specific findings are very rare, and the studies that have reached high positive predictive values of certain findings for abuse in infants have fundamental methodological flaws [32,65]. In the absence of satisfactorily empirical evidence from controlled studies, understanding the mechanism of the origin of the findings is essential. If the original SBS hypothesis is true, it would mean that the components of the triad are independent of each other and have a common traumatic cause in the form of shaking. This would mean that the presence of each component individually increases the probability of shaking. This reasoning is not valid if the components are dependent of each other, and the triad's specificity for shaking would then decrease.

For instance, if a re-bleeding in a chronic subdural hematoma is the first event in a pathophysiological chain leading to encephalopathy and retinal hemorrhage, it could be that shaking triggered the re-bleeding, but is could also be other kinds of trauma or even no trauma at all. Thus, if there is circumstantial evidence of shaking as the triggering event, you must be cautious when it comes to estimating the force of the shaking. Furthermore, there is a need to consider what might be the cause of the chronic component, e.g., is there a history of earlier blunt head trauma, evidence of benign external hydrocephalus or a possibility of birth related SDH.

The data of the present review also indicates that hypoxic brain injury, to some extent may be associated with isolated shaking. Although the underlying cause of this association could not be concluded from the information in the included cases, this result indicate that hypoxia may be a mediator of triad findings in infants subjected to shaking. If so, it is possible that other causes of hypoxia, e.g., spontaneous apnoea could end up with the same findings and that hypoxic brain injury in itself is inconclusive with respect to the underlying cause of triad findings. Lynoe & Eriksson have put forward the hypothesis that the starting point could be spontaneous apnea similar to apparent life threatening event (ALTE)/brief resolved unexplained event (BRUE), but with longer duration than in ALTE/BRUE [66]. In this model, you can hypothesize that the initial apnea is followed by other encephalopathy symptoms, e.g., seizures. It is also reasonable to assume shaking for the purpose of resuscitation may occur in such a scenario.

The result that 64 % of the final selection of 42 cases (both witnessed and admitted) had no other findings beside triad findings, indicates that absence of obviously independent traumatic findings such as bruises or fractures cannot be regarded as evidence of that shaking has not occurred.

Taken together, the forensic doctor needs to be cautious in the interpretation of triad findings and acknowledge that all suggested mechanisms are still hypothetical and in need for proper hypothesis testing. Furthermore, absence of findings indicative of abusive trauma that are independent of triad findings should not be interpreted as absence of a traumatic event.

### 4.4 Limitations

Although the definition of exposure used in this review should reduce the risk of introducing false positives, it does not eliminate it. In cases with admitted shaking, the confession appeared after the medical findings were detected. This methodological limitation has been highlighted in the study that contributed most cases to this review [47]. In another study, it is argued that admitted cases do not measure up as reference cases [67]. Some of the cases (n = 5) were from the USA, making it possible that some of the confessions were the result of a plea-bargain, meaning that in exchange for a confession the legal consequences could be lesser [68].

Another methodological limitation is that the identified cases can only show an association between the exposure and the findings, making it possible that this association could be about reverse causality, i.e., that the shaking was triggered by crying that in turn was a response to an ongoing intracranial process.

Mixed density subdural hematoma was assumed to signify re-bleeding in a chronic subdural hematoma in the present review. However, the term “mixed density”, sometimes referred to as “heterogeneous hematohygroma”, is descriptive and is believed to have four causes: 1) Mixture of fresh liquid blood and clots (estimated age range: 0–24 h), 2) Compact clot with serum separation (estimated age range: 1–3 days), 3) Mixture of fresh blood and CSF derived from vascular injury and arachnoid injury (estimated age range: 1 day–1 week), and 4) Fresh bleeding in chronic subdural hematoma (estimated age of chronic component at least 2 weeks) [69]. Thus, it could be that some of the mixed density cases were not cases having a re-bleeding in a chronic subdural hematoma. Due to the lack of details in the reporting of the subdural hematomas, this possibility could not be ruled out.

This overview is based on case series and case reports selected for both exposure and outcome. Thus, no conclusions can be drawn regarding the specificity or sensitivity of the selected findings to the exposure. Neither can conclusions be made about causality, since case reports lack control conditions. An exception is if there is an “internal control” in the form of a zero value regarding the outcome just before the exposure, i.e., negative neuroimaging/retinal examination before and in close connection to the exposure and positive findings directly after the exposure. No such case was identified.

### 4.5 Conclusion

Published case reports and case series on isolated shaking, selected for the presence of findings that often are considered as indicative of intentionally inflicted injury, lack information to allow for any conclusions that reaches above the hypothetical level regarding the mechanism of the findings. Understanding of the mechanism of the triad findings is key in the medico-legal setting, for which reason future studies focusing on the pathophysiology of various kinds of subdural hemorrhage and retinal hemorrhages are urgently needed.

## CRediT authorship contribution statement

**Ingemar Thiblin:** Conceptualization, Data curation, Formal analysis, Investigation, Methodology, Project administration, Resources, Validation, Writing – original draft, Writing – review & editing. **Carl Johan Wingren:** Conceptualization, Formal analysis, Investigation, Methodology, Writing – review & editing, Data curation, Validation. **Jacob Andersson Emad:** Conceptualization, Formal analysis, Software, Writing – review & editing. **Fredrik Tamsen:** Conceptualization, Data curation, Formal analysis, Investigation, Methodology, Validation, Writing – review & editing.

## Declaration of competing interest

The authors declare that they have no known competing financial interests or personal relationships that could have appeared to influence the work reported in this paper.

## Figures and Tables

**Table:** 

Variable	Outcome
Sex	Male/Female/Unclear
Age	In weeks
Country	
Information about trauma	Yes/No
Blunt force trauma head	Yes/No/Unclear/Not applicable (if no on Information about trauma)
Non-accidental blunt force head	Yes/No/Unclear/Not applicable (if no on Information about trauma)
Blunt force head before symptoms	Yes/No/Unclear/Not applicable (if no on Information about trauma)
Blunt force head after symptoms	Yes/No/Unclear/Not applicable (if no on Information about trauma)
Shaking	Yes/No/Unclear
Shaking before symptoms (crying not regarded as symptom)	Yes/No/Unclear
Shaking after debut of symptoms	Yes/No/Unclear
Findings indicative of blunt force head	Yes/No/Unclear
Skull fracture	Yes/No/Unclear/Not applicable (if no on Findings indicative of blunt force head trauma)
Bruise/soft tissue swelling/sratch mark/laceration head	Yes/No/Unclear/Not applicable (if no on Findings indicative of blunt force head trauma)
Shaking admitted before findings demonstrated	Yes/No/Unclear
Shaking admitted at hospital after findings demonstrated	Yes/No/Unclear
Shaking admitted during police interrogation or in cour	Yes/No/Unclear
Intracranial findings	Yes/No/Unclear
Subdural hematoma	Yes/No/Unclear/Not applicable (if no on Intracranial findings)
Acute subdural hematoma	Yes/No/Unclear/Not applicable (if no on Subdural hematoma)
Chronic subdural hematoma/hygroma	Yes/No/Unclear/Not applicable (if no on Subdural hematoma)
Mixed density subdural hematoma	Yes/No/Unclear/Not applicable (if no on Subdural hematoma)
Picture from neuradiology	Yes/No/Not applicable (if neuroradiology not performed)
Attenuation described (need not be figures; “acute”, “chronic”, “mixed density” accepted)	Yes/No/Not applicable (if CT not performed)
Information about subdural hematoma be unilateral or bilateral (neuroimaging or neuropathology)	Yes/No/Not applicable (If no on Subdural hematoma)
Information about thickness of subdural hematoma (neuroimaging or neuropathology)	Yes/No/Not applicable (If no on Subdural hematoma)
Information about expansive effect (neuroimaging or neuropathology)	Yes/No/Not applicable (If no on Subdural hematoma)
Subarachnoid hemorrhage	Yes/No/Unclear
Traumatic brain injury (if no information regarded as negative)	Yes/No
Hypoxic brain injury	Yes/No/Unclear
Brain edema (if no information regarded as negative)	Yes/No
Sinus thrombosis	Yes/No/Unclear
Thrombosis cortical vein	Yes/No/Unclear
Signs of benign external hydrocephalus (explicit statement wide subarachnoid spaces with normal or slightly expanded ventricles and/or rapid increase of skull circumference and or wide sutures	Yes/No/U nclear
Retinal hemorrhage	Yes/No/Unclear
Statement of retinal hemorrhage be extensive	Yes/No/Not applicable (If no or unclear on retinal hemorrhage)
Hemorrhage corpus vitreum	Yes/No/Unclear
Subhyaloid hemorrhage	Yes/No/Unclear
Retinoschisis	Yes/No/Unclear
CML	Yes/No/Unclear
Rib fracture	Yes/No/Unclear
Long bone shaft fracture	Yes/No/Unclear
Signs of raised intracranial pressure (objectively measured during surgery, subjective measure during surgery, e.g. “fluid emptying under high pressure in dural incision”, sun set gace, lateral eye deviation, tense/bulging fontanell)	Yes/No/Unclear
Bruise in other site than head (if clear that clinical examination was done, no comment regarded as No, with exception for articles focusing on only intracranial findings or eye findings; Unclear)	Yes/No/Unclear
Encephalopathy	Yes/No/Unclear
Seizures as first symptom	Yes/No/Unclear/Not applicable (in no or unclear on Encephalopathy)
Vomiting as first symptom	Yes/No/Unclear/Not applicable (in no or unclear on Encephalopathy)
Affected level of consciousness as first symptom	Yes/No/Unclear/Not applicable (in no or unclear on Encephalopathy)
Compromised breathing/apnea as first symptom	Yes/No/Unclear/Not applicable (in no or unclear on Encephalopathy)
Seizures on arrival at hospital	Yes/No/Unclear/Not applicable (in no or unclear on Encephalopathy)
Vomiting on arrival at hospital	Yes/No/Unclear/Not applicable (in no or unclear on Encephalopathy)
Affected level of consciousness on arrival at hospital	Yes/No/Unclear/Not applicable (in no or unclear on Encephalopathy)
Compromised breathing/apnea on arrival at hospital	Yes/No/Unclear/Not applicable (in no or unclear on Encephalopathy)
Prematurity	Yes/No/Unclear
Twin ship	Yes/No/Unclear
Small for gestational age	Yes/No/Unclear
Large for gestational age	Yes/No/Unclear
Sex perpetrator	Male/Female/Unclear
Time between shaking and debut of symptoms reported	Male/Female/Unclear/Not applicable (if no on Shaking)
Symptoms in direct connection with shaking	Yes/No/U (if yes on Time between shaking and debut of symptoms reported
Birth weight	Yes/no/Unclear
Information about deliviery	Yes/No
Neonatal disease	Yes/No/Unclear

**Table:** 

Serch number	Serch terms
1	Infant[MesH]
2	baby[Title/Abstract] OR babies[Title/Abstract] OR infanc∗[Title/Abstract] OR infant∗[Title/Abstract] OR neonat∗ [TitleAbstract] OR newborn∗[Title/Abstract] OR new-born∗[Title/Abstract] OR preterm∗[Title/Abstract] OR very young child∗[Title/Abstract]
3	1 OR 2
4	Hematoma, Subdural[MeSH] OR Brain Edema[MeSH] OR Brain injuries[MeSH] OR Eye Hemorrhage[MeSH] OR (Hemorrhage[MeSH:NoExp] AND Brain[MeSH:NoExp] OR Eye[MeSH:NoExp]) OR Fractures, Bone[Mesh:NoExp] OR Tibial Fractures[Mesh] OR Epiphyses/injuries[MeSH] OR Rib Fractures[MeSH]
5	subdural hemorrhage∗[Title/Abstract] OR subdural hemorrhage [Title/Abstract] OR subdural hematoma∗[Title/Abstract] OR subdural hematoma∗[Title/Abstract] OR retinal hemorrhage∗[Title/Abstract] OR retinal hemorrhage∗[Title/Abstract] OR intraocular bleeding∗[Title/Abstract] OR intraocular hemorrhage∗ [Title/Abstract] OR intraocular hemorrhage∗ [Title/Abstract]OR vitreous hemorrhage∗[Title/Abstract] OR vitreous hemorrhage∗ OR brain swelling∗[Title/Abstract] OR cerebral edema∗[Title/Abstract] OR cerebral edema∗[Title/Abstract] OR brain edema∗[Title/Abstract] OR brain edema∗[Title/Abstract] OR rib fracture∗[Title/Abstract] OR long bone fracture∗[Title/Abstract] OR Classic Metaphyseal Lesion[Title/Abstract] OR metaphyseal fracture∗[Title/Abstract] OR metaphyseal chip fracture∗[Title/Abstract] OR tibial fracture∗ [Title/Abstract] OR brain injur∗[Title/Abstract] OR traumatic brain injur∗ [Title/Abstract] OR cerebral swelling∗ [Title/Abstract] OR retinal bleeding∗[Title/Abstract]
6	4 OR 5
7	Accidents[MeSH] OR Accidental Falls[MeSH] OR Child Abuse[MeSH] OR Diagnosis, Differential[MeSH] OR Diagnostic Errors[MeSH] OR Multiple Trauma[Mesh]
8	accident∗[Title/Abstract] OR abus∗[Title/Abstract] OR differential diagnos∗[Title/Abstract] OR inflicted[Title/Abstract] OR intentional[Title/Abstract] OR misdiagnosed[Title/Abstract] OR nonaccident∗[Title/Abstract] OR non-accident∗[Title/Abstract] OR noninflicted[Title/Abstract] OR non-inflicted[Title/Abstract] OR odds of abuse[Title/Abstract]OR presumptive[Title/Abstract] OR probable[Title/Abstract] OR suspect∗[Title/abstract] OR underdiagnosed[Title/Abstract] OR unintentional[Title/Abstract]
9	7 OR 8
10	Shaken Baby Syndrome[Mesh]
11	abusive head injur∗[Title/Abstract] OR abusive head trauma∗[Title/Abstract] OR battered child syndrome∗[Title/Abstract]OR infant head trauma∗[Title/Abstract] OR infant tbi[Title/Abstract] OR shaken[Title/Abstract] OR shaking[Title/Abstract] OR pediatric tbi[Title/Abstract]
12	10 OR 11
13	6 AND 9
14	12 OR 13
15	3 AND 14
16	Filter: English

((Infant[MesH]) OR (baby[Title/Abstract] OR babies[Title/Abstract] OR infanc∗[Title/Abstract] OR infant∗[Title/Abstract] OR neonat∗ [Title/Abstract] OR newborn∗[Title/Abstract] OR new-born∗[Title/Abstract] OR preterm∗[Title/Abstract] OR very young child∗[Title/Abstract])) AND (((Shaken Baby Syndrome[Mesh]) OR (abusive head injur∗[Title/Abstract] OR abusive head trauma∗[Title/Abstract] OR battered child syndrome∗[Title/Abstract]OR infant head trauma∗[Title/Abstract] OR infant tbi[Title/Abstract] OR shaken[Title/Abstract] OR shaking[Title/Abstract] OR pediatric tbi[Title/Abstract])) OR (((Hematoma, Subdural[MeSH] OR Brain Edema[MeSH] OR Brain injuries[MeSH] OR Eye Hemorrhage[MeSH] OR (Hemorrhage[MeSH:NoExp] AND Brain[MeSH:NoExp] OR Eye[MeSH:NoExp]) OR Fractures, Bone[Mesh:NoExp] OR Tibial Fractures[Mesh] OR Epiphyses/injuries[MeSH] OR Rib Fractures[MeSH]) OR (subdural hemorrhage∗[Title/Abstract] OR subdural hemorrhage [Title/Abstract] OR subdural hematoma∗[Title/Abstract] OR subdural hematoma∗[Title/Abstract] OR retinal hemorrhage∗[Title/Abstract] OR retinal hemorrhage∗[Title/Abstract] OR intraocular bleeding∗[Title/Abstract] OR intraocular hemorrhage∗ [Title/Abstract] OR intraocular hemorrhage∗ [Title/Abstract]OR vitreous hemorrhage∗[Title/Abstract] OR vitreous hemorrhage∗ OR brain swelling∗[Title/Abstract] OR cerebral edema∗[Title/Abstract] OR cerebral edema∗[Title/Abstract] OR brain edema∗[Title/Abstract] OR brain edema∗[Title/Abstract] OR rib fracture∗[Title/Abstract] OR long bone fracture∗[Title/Abstract] OR Classic Metaphyseal Lesion[Title/Abstract] OR metaphyseal fracture∗[Title/Abstract] OR metaphyseal chip fracture∗[Title/Abstract] OR tibial fracture∗ [Title/Abstract] OR brain injur∗[Title/Abstract] OR traumatic brain injur∗ [Title/Abstract] OR cerebral swelling∗ [Title/Abstract] OR retinal bleeding∗[Title/Abstract])) AND ((Accidents[MeSH] OR Accidental Falls[MeSH] OR Child Abuse[MeSH] OR Diagnosis, Differential[MeSH] OR Diagnostic Errors[MeSH] OR Multiple Trauma[Mesh]) OR (accident∗[Title/Abstract] OR abus∗[Title/Abstract] OR differential diagnos∗[Title/Abstract] OR inflicted[Title/Abstract] OR intentional[Title/Abstract] OR misdiagnosed[Title/Abstract] OR nonaccident∗[Title/Abstract] OR non-accident∗[Title/Abstract] OR noninflicted[Title/Abstract]OR non-inflicted[Title/Abstract] OR odds of abuse[Title/Abstract]OR presumptive[Title/Abstract] OR probable[Title/Abstract] OR suspect∗[Title/abstract] OR underdiagnosed[Title/Abstract] OR unintentional[Title/Abstract])))).

**Table:** 

Reference	Insufficient data	Not witnessed or admitted	Not shaking	Shaken after onset of symptoms	Unclear when shaking occurred	Review or Guidelines	No intracranial finding	Evidence of blunt head trauma
[70]		1						
[71]		1						
[72]		1						
[73]								1
[74]		1						
[75]		1						
[76]		1						
[77]					1			1
[78]		1						
[79]					1			
[80]								1
[81]		1						
[82]				1	1			
[83]		1						
[84]						1		
[85]							1	
[86]			1					
[87]		1						
[88]					1			
[89]			1					
[90]							1	
[91]								1
[92]								1
[93]	1	1						
[94]	1							
[95]	1				1			
[96]		1						
[97]					1			
[98]				1	1			
[99]		1	1					1
[100]	1	1						
[101]		1						
[102]		1						
[13]	1							
[14]	1							
[103]		1						
[104]	1							
[105]					1			1
[106]	1				1			
[107]	1				1			1
[108]		1						
[109]		1						
[110]		1		1				
[111]		1						
[112]		1						
[113]		1						
[114]								1
[115]		1						
[116]		1						
[117]					1			1
[118]		1						
[119]		1						
[120]	1							
[121]		1						
[122]		1						
[123]	1							
[124]						1		
[125]		1						
[126]		1						
[127]		1						
[128]		1						
[129]		1	1					
[130]		1						
[131]	1							
[132]	1	1						
[133]						1		
[134]	1				1			
[135]		1						
[136]					1			
[137]		1						
[138]			1					
[139]			1					
[140]			1					
[141]								1
[142]		1						
[143]		1						
[144]								1
[145]		1						
[37]	1							
[146]		1						
[147]	1			1				
[148]		1			1			
[149]	1							
[150]								
[151]	1	1						
[152]		1						
[153]		1						
[154]								1
[155]	1							
[156]	1							
[157]	1				1			
[158]		1					1	
[159]		1						
[160]					1			
